# A Longitudinal Study on the Relations Among Fear-Enhancing Parenting, Cognitive Biases, and Anxiety Symptoms in Non-clinical Children

**DOI:** 10.1007/s10578-019-00868-7

**Published:** 2019-02-14

**Authors:** Lorraine Fliek, Jeffrey Roelofs, Gerard van Breukelen, Peter Muris

**Affiliations:** 1 Department of Clinical Psychological Science, Faculty of Psychology and Neuroscience, Maastricht University, P.O. Box 616, 6200 MD Maastricht, The Netherlands; 2 Department of Methodology & Statistics, Faculty of Psychology and Neuroscience & CAPHRI School for Care and Public Health, Maastricht University, Maastricht, The Netherlands; 3 Stellenbosch University, Stellenbosch, South Africa

**Keywords:** Children’s anxiety symptoms, Cognitive biases, Parenting, Modeling, Threat information transmission.

## Abstract

This longitudinal study explored the relations between fear-enhancing parenting behaviors (modeling and threat information transmission) and children’s cognitive biases and anxiety symptoms on three subsequent time points over a one-year period. Participants were 216 children aged 7–12 years (114 boys and 102 girls), and their mothers (*n* = 199) and/or fathers (*n* = 117). On each time point, children and parents completed the Parental Enhancement of Anxious Cognitions scale, which measures parental modeling and threat information transmission. Furthermore, children filled in a measure of anxiety disorder symptoms. In addition, confirmation bias and interpretation bias were measured by means of a number of computerized tasks. The results yielded support for a circular model in which cognitive biases enhanced anxiety symptoms, which in turn promoted cognitive biases on each of the three time points. However, no evidence was found for longitudinal effects of cognitive biases on anxiety or vice versa. In contrast to what we expected, cognitive biases and anxiety appeared to promote parental modeling and threat information rather than the other way around. These findings extend research on the relations between parenting behaviors, cognitive biases, and childhood anxiety symptoms, and suggest valuable leads for assessment and intervention.

Epidemiological studies show that anxiety disorders are among the most common mental disorders in childhood [1–3]. Given their high prevalence rates, research on the etiology and maintenance of anxiety disorders in children and adolescents is important and it is good to see that in previous decades considerable advancements have been made in our understanding of the factors involved in the pathogenesis of this type of psychopathology (see reviews by [4, 5]). According to Beck’s [6] cognitive theory, information processing biases play an important role, and there is indeed evidence showing that these biases are involved in the maintenance and exacerbation of anxiety problems [7]. It is also known that anxiety-related cognitive biases not only occur in adults but are also present in children [8, 9].

Anxiety-related cognitive biases are concerned with overactive schemas involving the themes of threat and danger [7], which manifest themselves as cognitive distortions that occur during subsequent stages of information processing [10]. In the early stages, where information about the internal or external world is automatically perceived and encoded, anxious individuals display a typical tendency to shift their attention towards threatening stimuli (i.e., attentional bias). During later stages, information is consciously interpreted and transformed into action tendencies and actual behavior. Two types of cognitive biases in these later stages have our special interest in the present investigation. The first one is interpretation bias, which can be defined as the tendency to interpret ambiguous stimuli and situations as threatening [11]. The most widely used method for assessing this type of bias is the ambiguous vignette paradigm, which makes use of a series of short descriptions of everyday situations that may occur in daily life. Children are asked to score the perceived level of threat or indicate how they would respond in such situations. From both types of responses one can reliably infer whether children interpret the ambiguous scenarios as dangerous, a tendency that has been demonstrated to be present in high-anxious non-referred children [12–14] as well as in children with anxiety disorders [15, 16]. The second cognitive distortion is confirmation bias, which refers to the selective search for information that concurs with the view that one holds, while ignoring information that might disconfirm this view [11]. A paradigm that is often employed to investigate confirmation bias is a task in which children are given the opportunity to select additional, negative or positive information about a novel (potentially) threatening stimulus (often an unknown animal) or situation. It has been shown that anxious children display a stronger tendency to search for negative information and less frequently tend to choose positive information, indicating the presence of a confirmation bias [17–20].

Like other psychopathological phenomena, the origins of anxiety-related cognitive biases are assumed to be due to genetic and environmental factors, which also interact with each other [8]. There is evidence showing that cognitive biases occurring during the early stages of information processing (i.e., attentional biases) are more clearly associated with genetic factors such as an inherited liability in the serotonin-transporter gene [21, 22]. Meanwhile, the contribution of heritability to anxiety-related distortions that take place during the later stages of processing (such as interpretation bias and confirmation bias) has been shown to be quite modest [23]. For these biases, it seems more plausible to investigate the role of environmental factors, of which familial influences such as parenting can be considered as especially relevant. Murray, Creswell, and Cooper [24] have outlined a cognitive-behavioral model in which parents’ distorted cognitions (e.g., threat interpretation bias) and in its wake expectations of the child (about how well it can control or cope with [potentially] dangerous situations) fuel fear-enhancing parenting behaviors such as modeling (i.e., a parent showing fear and anxiety reactions in the presence of the child [25]) and verbal threat information transmission (i.e., a parent verbally expressing to the child that a stimulus or situation might be dangerous [26]), which ultimately will install anxious information processing biases in the child (see also [27]).

The support for a scenario in which parenting behaviors promote cognitive biases and subsequent anxiety symptoms in children has been mainly circumstantial [28]. That is, research so far demonstrated that: (1) anxious parents have anxious children [29]; (2) anxious parents display threat-related cognitive biases that tend to generalize to their child’s environment [30] and that may also install similar cognitive biases in offspring [31]; and (3) the parenting behaviors of modeling and verbal threat information transmission are involved in the transfer of anxiety and threat-related biases from parents to children [32, 33].

Attempts to investigate the relations among parenting, cognitive bias, and anxiety in children in one and the same study are relatively sparse. One exception is an investigation by Barrett et al. [34] who assessed interpretation bias in anxious youths aged 7–14 years and explored how family processes influenced children’s interpretations of ambiguity. Clinically referred children with anxiety disorders (*n* = 152), children with externalizing problems (i.e., oppositional-defiant disorder; *n* = 27), and non-clinical control children without any problems (*n* = 26) and their parents were asked separately to interpret and provide action plans for a series of ambiguous scenarios. Following this initial assessment, children and parents were asked to discuss a number of these scenarios together, after which children were invited to provide a final response for each of the scenarios. The results indicated that the anxious and aggressive children were both more likely to interpret the ambiguous scenarios in a threatening way as compared to the non-clinical control group, with anxious children more often choosing avoidant action plans and oppositional-defiant children more frequently selecting aggressive solutions. Most importantly, the family discussions strengthened this pattern of results, showing significant increases in anxious children’s avoidant action plans and oppositional-defiant children’s aggressive action plans. Although these findings are relevant for multiple types of psychopathology, they clearly demonstrate that in the case of anxiety problems, a threat-related cognitive style may develop within the context of anxiety-enhancing family processes.

A recent study by Sicouri et al. [35] used a similar procedure to explore the relations between parent–child discussions, cognitive bias, and anxiety in children with asthma. Eighty-nine parent–child dyads were included across four groups: children with asthma and anxiety (*n* = 29), children with anxiety only (*n* = 21), children with asthma only (*n* = 15), and healthy control children (*n* = 24), all aged between 8 and 13 years. Interpretation bias was measured using two types of scenarios related to general threat and asthma threat. It was found that children with anxiety displayed an interpretation bias in response to the general threat scenarios, whereas children with asthma exhibited an interpretation bias in response to the asthma threat scenarios. Most interestingly, following parent–child discussions, changes in avoidance reactions were noted, and these mainly occurred in the anxious children in response to asthma threat scenarios.

Another study was conducted by Remmerswaal, Muris, and Huijding [36] who explored the role of parents in the development of a cognitive bias and subsequent fear levels in non-clinical children aged between 8 and 13 years who were confronted with a novel animal. More precisely, in two experiments (*N*’s being 122 and 49), it was examined whether instructed (experiment 1) or spontaneous (experiment 2) verbal feedback of mothers induced a negative information search strategy (i.e., confirmation bias) in their offspring. The results convincingly demonstrated that the verbal feedback of the mothers (either provided on instruction or given spontaneously—based on their own cognitive distortion) had a significant impact on children’s cognitive bias. More precisely, when mothers verbally encouraged their offspring to search for threat-related information about the animal, children indeed displayed a stronger confirmation bias, which subsequently also resulted in increased fear for the unknown animal. Again, this can be taken as supportive evidence for the role of parenting variables in the intergenerational transmission of cognitive biases from mothers to children [36].

In further research by Van Niekerk et al. [37], anxiety-related interpretation biases were measured in 7- to 14-year-old children of whom the parents were diagnosed with panic disorder (PD; *n* = 44), social anxiety disorder (SAD; *n* = 27), comorbid PD and SAD (*n* = 7), or of whom parents did not suffer from an anxiety disorder at all (*n* = 84). A set of ambiguous scenarios with a panic or social threat content was used, which had to be interpreted by the children under two conditions: first without priming and one week later with priming (i.e., viewing a video of an adult telling what it is like to have a specific anxiety disorder). The results indicated that in general children’s own level of anxiety symptoms was predictive of interpretation bias scores. Parental anxiety diagnosis also played a significant role: more precisely, it was found that children of parents with PD, but not the offspring of parents with SAD, displayed a stronger tendency to interpret ambiguous scenarios in a more negative way as compared to children of parents without anxiety disorders. Priming appeared not to have a significant impact on children’s interpretation of the scenarios. A similar study by Schneider, Unnewehr, Florin, and Margraf [38], which was conducted more than 15 years earlier, compared 29 children of parents with PD, 21 children of parents with animal phobias, and 30 children of parents without a mental disorder (all children were between 8 and 15 years old). Findings revealed that children of parents with PD, but not those of parents with animal phobia or healthy control parents, showed an increase in anxious interpretations, although in this study the effect could only be demonstrated when children had been primed with a panic-relevant model. Although the priming procedure used in both studies [37, 38] certainly bears resemblance to the modeling and verbal threat transmission phenomena that have been described earlier, it should be borne in mind that the use of a video model makes these investigations less relevant if one wants to gain insight in the family processes underlying the transfer of cognitive biases and anxiety from parents to children. Nevertheless, the findings do confirm that there are significant links between parental anxiety (especially in case symptoms are clearly noticeable as in PD) and children’s cognitive biases and anxiety symptoms.

While the studies by Barrett et al. [34], Schneider et al. [38], Remmerswaal et al. [36], Sicouri et al. [35], and Van Niekerk et al. [37] were all to some extent experimental in nature, Fliek et al. [39] relied on a different research approach. They conducted a cross-sectional survey to examine the relations between parenting, cognitive bias, and anxiety symptoms in a sample of 258 children aged 7 to 12 years. Interestingly, the fear-promoting parenting behaviors of modeling and threat information transmission were both measured by means of a specifically construed scale in fathers as well as mothers. Further, children’s cognitive distortions of interpretation bias and confirmation bias and DSM-defined anxiety disorder symptoms were also assessed. The results indicated that both types of cognitive biases mediated the relationship between threat information transmission (of both parents) and children’s anxiety symptoms, while only interpretation bias significantly mediated the relationship between modeling (of mothers) and children’s anxiety symptoms.

The data of the Fliek et al. [39] study were collected as part of a longitudinal study on the relations between cognitive biases, anxiety disorder symptoms, and family factors that might influence the relation between these variables. Follow-up assessments have now been conducted in this sample of children on two further time points (i.e., after 6 and 12 months) and these data will be included in the present study, so that it becomes possible to investigate the relations among fear-enhancing parenting, cognitive bias, and anxiety symptoms in children longitudinally. Thus, the fear-enhancing parental variables of modeling and verbal threat information transmission, the two types of cognitive biases: interpretation bias and confirmation bias, and anxiety symptoms were measured in 258 children aged 7 to 12 years on three occasions. The data were used to test the following hypotheses: First of all, with regard to the relations between cognitive biases and children’s anxiety symptoms on a cross-sectional level, we predicted to find support for a bidirectional (circular) model [17] in which cognitive biases promote anxiety symptoms, and anxiety symptoms in turn enhance cognitive biases. Second, we expected that children’s cognitive biases and anxiety symptoms would be stable over time. A third hypothesis concerned longitudinal effects, and implied that cognitive biases would enhance children’s anxiety symptoms on subsequent time points, which of course would substantiate the idea that these distortions are involved in the maintenance and exacerbation of anxiety problems [6, 7], but also that anxiety problems would promote cognitive distortions over time, which is in keeping with the abovementioned bi-directional model. As a fourth hypothesis, we predicted that detrimental parenting behaviors would lead to more cognitive distortions and higher anxiety levels, both on a cross-sectional and on a longitudinal level. Fifth and finally, based on the most current theoretical notions and the findings of previous studies [28, 34, 36, 39], we expected to find support for a longitudinal mediational model in which the cognitive distortions of interpretation bias and confirmation bias mediate the relation between the parenting behaviors of modeling and threat information transmission on the one hand, and children’s anxiety symptoms on the other hand.

With regard to the cross-sectional and longitudinal model testing, it is important to note the current data were also used to test a number of (plausible) alternative models, since it should not only be proven that a hypothesized model fits the data well, but also that it fits *better* than alternative models. For instance, we examined the fit of models in which both biases were correlated versus models in which one bias affected the other, and a model in which anxiety symptoms and both cognitive biases were all correlated to each other versus models with bias affecting anxiety or vice versa. Further, in our exploration of parental influences, we also tested a model in which anxiety symptoms and cognitive biases promoted detrimental parenting, as there is some evidence in the literature that when children are highly anxious, their parents become more overprotective (and thus likely show modeling and threat information transmission) [40].

## Method

### Participants

At the beginning of the study, the sample consisted of 258 non-clinical children (132 boys and 126 girls) aged between 7 and 12 years (*M* = 9.52, *SD* = 1.38). All children had the Dutch nationality and the majority of them were Caucasian (> 95%). The remainder of the families originated from diverse ethnic backgrounds (e.g., North African, Arabic, Asian). Questionnaires about parental behaviors nearly always referred to children’s biological parents; the two exceptions being one child who had adoptive parents and one child who was raised by his mother and her female partner. The latter child only answered the questions with regard to the biological mother. About 15% of the children came from divorced families. For the ultimate analysis, we only used those cases for which we had complete child data on each of the three time points. More precisely, on the second time point, four children dropped out, whereas on the third time point, another 38 children dropped out, and as a result the final sample of participants consisted of 216 children (114 boys and 102 girls). Independent-samples *t*-tests revealed that children who dropped out (*M* = 10.99, *SD* = 1.07) were significantly older than children who did not drop out (*M* = 9.69, *SD* = 1.14; *t*(256) = 6.76, *p* < .001), whereas they did not differ significantly with regard to anxiety, both types of cognitive biases (all *t*(256)’s < 1, *p*’s > 0.05), and gender (*χ*^2^(1) = 1.12, *p* > 0.05) as measured at baseline.^1^

Parents were also asked to fill out the questionnaire measuring modeling and threat information transmission. A total of 199 mothers and 117 fathers (mean ages being 42.20 years, *SD* = 4.42 and 44.36 years, *SD* = 4.95, respectively, range 28–65 years) did so on the first time point of this study. On the second time point, 18% of mothers and 14% of fathers dropped out. On the third time point, another 6% of mothers as well as 6% of fathers dropped out. Independent-samples *t*-tests revealed no significant differences between parents who dropped out and parents who did not drop out for any of the child and parent variables that were assessed in this study (all *t*(df)’s ≤ 1.98, *p’*s > 0.05), where df varied from 79 to 174 depending on the variable for which dropouts were compared to non-dropouts, and depending on the different sample sizes for mothers and fathers. For the statistical analyses of the parent data, we only used those cases for which we had obtained data on all three time points (*N* = 147). For 43 children only the mother completed all three assessments. For three children only the father completed the three assessments. For a total of 44 children, the three assessments were completed alternately by either the mother or the father. For the remaining 57 children, both parents completed all three assessments and their scores were averaged for the final data analysis. As compensation for their participation, families, for whom children and parents had completed questionnaires on all three assessment points, received a set of cinema vouchers (which each had a value of 7.50 Euro).

### Measures

The *parental enhancement of anxious cognitions* (PEAC) was used to assess parental modeling and threat information transmission behaviors. The scale was construed for the purpose of this research project [39]. The PEAC asks children to rate the frequency of parents’ fear-enhancing behaviors, using a 4-point Likert scale (0 = never, 1 = sometimes, 2 = often, and 3 = always). The questionnaire consists of 14 items: 6 items are concerned with modeling behaviors of parents (e.g., “This parent shows me that he/she is afraid to do certain things”), while 8 items have to do with threat information transmission (e.g., “This parent warns me explicitly that I should avoid dangerous situations”). Each item is answered twice, once for the mother and once for the father. For each parent, PEAC modeling and threat information transmission scores are computed by summing the ratings on relevant items. In the current study, Cronbach’s alphas of the modeling scale for mothers and fathers varied between 0.65 and 0.84 on various assessment points, while alphas of the threat information scale ranged between 0.80 and 0.92. These values indicate that the PEAC scales have sufficient to good reliability in terms of internal consistency.

The *parent version of the PEAC* (PEAC-P) is similar to the questionnaire that is completed by the children, but the 14 items are formulated from the perspective of the parent (e.g., “I warn my child explicitly that he/she should avoid dangerous situations” instead of “This parent warns me explicitly that I should avoid dangerous situations”). Cronbach’s alphas of the PEAC-P modeling scale for mothers and fathers varied between 0.63 and 0.78, while alphas of the threat information scale ranged between 0.73 and 0.83. These values indicate that the internal consistency of the PEAC-P is similar to that of the child version.

The *Revised version of the Screen for Child Anxiety Related Emotional Disorders* (SCARED-R; [41]) is an extension of the original SCARED [42]. The scale assesses symptoms of the entire spectrum of DSM-IV-defined anxiety disorders [43, 44]. In the current study only the SCARED-R subscales of social phobia (7 items; e.g., “I don’t like to be with unfamiliar people”), generalized anxiety disorder (9 items; e.g., “I worry about things working out for me”), and separation anxiety disorder (8 items; e.g., “I don’t like being away from my family”) were used, because these three types of anxiety were considered as most relevant in relation to the scenarios that were employed to assess cognitive biases (see below). Children were asked to rate the frequency with which they experienced each symptom using a three-point scale (0 = almost never, 1 = sometimes, 2 = often) and a total anxiety score was obtained by summing ratings on the items of the three selected anxiety scales (range 0–48). Research has demonstrated that the SCARED-R has good internal consistency, test–retest reliability, and validity [41, 45, 46]. In the present study, the internal consistency of the SCARED-R total anxiety score was excellent, with Cronbach’s alphas of 0.87, 0.88 and 0.90 on the three successive time points.

The *information search task* (IST) was based on the paradigm used by Remmerswaal et al. [17] and adjusted for the purpose of the current study to assess children’s confirmation bias. The adjustments to the IST entailed that the stories described real-life situations that reflected social, separation, and general anxiety themes instead of stories involving a novel animal, which were employed by Remmerswaal et al. [17]. Children were presented with new, potentially threatening situations (e.g., going to a new school) about which they had to gain additional information (e.g., “What would you like to know about the teachers at your new school?”) by choosing between a positive (e.g., “Whether they have a nice way of teaching”) and a negative (e.g., “Whether they become angry very easily”) option. Following their choice, children always received a confirming response (e.g., positive: “Most teachers have a nice way of teaching”, negative: “Most teachers become angry very easily”). In total, children were presented with three situations (one per anxiety theme) on each time point, for each of which they were given five opportunities to gain extra information. A confirmation bias score was computed for each time point by summing the number of negative options chosen (range 0–15). The reliability in terms of internal consistency of the IST proved to be satisfactory in this study, with Cronbach’s alphas of 0.75, 0.81, and 0.80 on the three time points.

*Ambiguous stories* [13, 47] were used to assess interpretation bias. These stories represented the themes of social anxiety (e.g., going to a sporting club for the first time), generalized anxiety (e.g., driving with your bike on a very busy street), and separation anxiety (e.g., staying with a friend while parents are on vacation). Children had to read the stories, which consisted of five sentences presented to them sentence by sentence on the computer screen. An example of a story relating to the theme of generalized anxiety would be: “You ride on the bike slowly because you are carrying a large bag with purchases. You ride on a street without a bikeway. It is a very busy street. The cars that pass you by drive very fast. You hear a big truck approaching from behind (see [13, 47]). Following each sentence, children were asked whether they thought that the story would ultimately be “scary” or “not scary”. On each time point, children were presented with three different stories (again one for each of the three anxiety themes). A total interpretation bias score was calculated for each time point by summing the number of sentences after which children indicated that the story was going to be scary (range 0–15). The reliability in terms of internal consistency of the Ambiguous Stories test was good, with Cronbach’s alphas of 0.79, 0.81, and 0.81 on the three consecutive time points.

### Procedure

Participants were recruited via four primary schools in the Southern part of The Netherlands. Informed parental consent was obtained by sending parents an information letter about the study along with a consent form. Children for whom parents granted permission were tested in small groups (of approximately eight children per group) in a separate room at school. Each child used a computer to fill out the questionnaires (PEAC and SCARED-R) and to conduct the cognitive bias tasks. This assessment took place under supervision of two experimenters, who guided the children through the session by providing instructions and by collectively conducting some practice items. Children received explicit instructions to call upon the experimenters in case they had any questions about the scales or tasks. The experimenters remained always present during the assessments to ensure that the children worked independently and completed all questionnaire and test items. Children first completed the SCARED and the PEAC, after which they carried out the IST and the Ambiguous Stories test. Parents completed the questionnaire at home on their own computer using a web-link provided to them by the experimenters. Follow-up assessments were conducted on two time points, 6 and 12 months after the initial assessment session. Children who were in the last grade of primary school when the study started, had already left school at the time that the 12 month follow-up assessment took place. These children received an e-mail that included an internet link which enabled them to carry out the final assessment at home.

### Statistical Analyses

Descriptive statistics and correlations were computed to inspect cross-sectional and longitudinal links among all variables that were measured in the study. Further, the presence of differences among the four participating schools with respect to the mean scores of various variables on the three time points was checked by repeated measures analyses of variance (ANOVAs), with school as between-subject factor and time as within-subject factor. Data were further analyzed by means of structural equations modeling using LISREL 8.80 [48, 49]. For all children the child-reported PEAC data of the mother and father were averaged, and this procedure seemed justified as scores for both parents were substantially correlated (i.e., *r*’s were respectively 0.70 for modeling and 0.83 for threat information transmission on time point 1, 0.60 and 0.80 on time point 2, and 0.57 and 0.78 on time point 3, all *p*’s < 0.001). Further, preliminary regression analyses showed that child-reported mother and father PEAC scores displayed similar relations with children’s cognitive biases and anxiety symptoms. Confirmation and interpretation bias were analyzed separately in the model, since they were not particularly strongly correlated (*r* = 0.20 on time point 1, *r* = 0.28 on time point 2, and *r* = 0.36 on time point 3, all *p*’s < 0.01). Moreover, it can be assumed that there is a logical temporal order for both biases, with interpretation bias being the starting point of conscious threat perception and confirmation bias constituting a cognitive response occurring once threat has been perceived. Age and sex were included as exogenous variables in all models as previous research has indicated that these may have an effect on childhood anxiety symptoms (and possibly related psychological constructs): that is, anxiety symptoms tend to decline as children become older and girls generally display higher levels of anxiety symptoms than boys [50–52].

A four-step procedure was followed for the LISREL analyses. First, we conducted a series of analyses to explore the direction of the relations between child anxiety, confirmation bias, and interpretation bias on each of the three time points. To this end we tested a number of theoretically plausible models: First, we tested a model with arrows pointing from both (correlated) biases to anxiety (model 1; see Table 3 of the results section for a graphical overview of all the models). We also tested a model with arrows pointing in the opposite direction (model 2), which essentially assumes that both (correlated) biases are the result of anxiety. Another plausible model that was tested was a circular model, since we know from previous research that cognitive bias and anxiety influence each other reciprocally [17]. In this circular model (model 3) interpretation bias was placed before confirmation bias, since—as noted above—interpretation and perception of threat is likely to occur before one can search for confirming or disconfirming information. Another model resembled model 1, by assuming that anxiety is the result of both biases, but here interpretation bias preceded confirmation bias (model 4). In a similar vein, a further model was tested as a variant of model 2, with both biases being the result of anxiety and interpretation bias preceding confirmation bias (model 5). A final model hypothesized that anxiety, interpretation bias, and confirmation bias were all inter-correlated with no clear-cut directions among these three variables (model 6). All models were tested twice, once without and once with equality constraints. Testing without equality constraints means that every causal path at every time point has its own regression weight, whereas in the analyses with equality constraints it was assumed that the path coefficients were stable over the three time points for any given causal path, such as from interpretation bias to anxiety (thereby reducing the number of path coefficients and making the model more parsimonious). In each of these models a residual correlation was included between the three repeated measures of the same variable (e.g., between anxiety at time 1, 2 and 3).

As a second step, the best fitting model was tested longitudinally by adding time-lagged paths between the three variables across the three time points, for instance between cognitive biases on one time point (t) and anxiety symptoms on a subsequent time point (t + 1) and between anxiety symptoms on t and biases on t + 1. Third, the parenting variables (PEAC modeling and threat information transmission) were added in order to test the cross-sectional relations between the parenting variables on the one hand and anxiety and both biases on the other hand. Fourth, again the best fitting model was tested longitudinally by adding time-lagged paths between parenting and child variables. Further details of all models will be given in the “Results” section.

Model fit was assessed by means of the following goodness-of-fit indices: the Chi Square test, the root mean square of approximation (RMSEA), the normed fit index (NFI), the non-normed fit index (NNFI), and the comparative fit index (CFI). Apart from the first measure, all measures combine goodness of fit (Chi Square) with model parsimony (degrees of freedom). For the RMSEA, lower values are indicative of a better fit and values of 0.05 and 0.08 can be considered as respectively good and reasonable [53]. The NFI, NNFI, and CFI range between 0 (poor) and 1 (excellent), and for these indices values thus need to be large, with 0.90 being the cut-off for defining a good fit.

## Results

### Mean Differences and Correlations Among Parenting, Biases, and Anxiety

Table 1 displays the mean scores and standard deviations for various child-completed measures as obtained on the three time points as well as the cross-sectional and longitudinal correlations among these variables. Apart from the finding that longitudinal associations between different variables were in general weaker than cross-sectional relations, a number of conclusions can be drawn from this table. To begin with, test–retest correlations varied between 0.56 and 0.70 (*p*’s < 0.01) for anxiety symptoms, 0.42 and 0.61 (*p*’s < 0.01) for confirmation bias, 0.42 and 0.50 (*p*’s < 0.01) for interpretation bias, 0.41 and 0.50 (*p*’s < 0.01) for parental modeling, and 0.44 and 0.55 (*p*’s < 0.01) for parental threat information transmission. Repeated measures ANOVAs showed that there was no effect of school for any of the variables (all *p*’s > 0.05). A significant time effect was found with regard to anxiety scores [*F*(2, 430) = 10.28, *p* < .001, ηρ^2^ = 0.05]. Post-hoc tests showed that children’s anxiety scores significantly decreased from time point 1 to time point 2 (*p* = 0.01), with no significant change being observed from time 2 to time 3 (*p* = 0.11). A repeated measures ANOVA for confirmation bias also revealed a significant time effect [*F*(2, 430) = 32.91, *p* < 0.001, ηρ^2^ = 0.13]. Post-hoc tests revealed a significant decrease from time 1 to time 2 (*p* < 0.001), while there was a significant increase from time 2 to time 3 (*p* < 0.001). The repeated measures ANOVAs with interpretation bias, parental modeling, and threat information transmission as dependent variables did not show significant time effects (all *p*’s > 0.05). Further, it was found that children’s anxiety symptoms were significantly correlated with cognitive biases and parenting variables on all three time points (*r*’s between 0.30 and 0.51, *p*’s < 0.01). Finally, although cognitive biases were as expected positively associated with modeling and threat information transmission, these correlations were modest and sometimes even non-significant (*r*’s between 0.10, *p* > 0.05 and 0.29, *p*’s < 0.01).

**Table 1:** Means and standard deviations for all child-completed measures as obtained on the three time points of the study, as well as correlations among these variables

	*M*	*SD*	(1)	(2)	(3)	(4)	(5)	(6)	(7)	(8)	(9)	(10)	(11)	(12)	(13)	(14)
Time 1																
1. SCARED-R Anxiety	16.86	8.47														
2. IST C-bias	8.99	3.41	0.32**													
3. Amb Stories I-bias	5.24	3.29	0.51**	0.18**												
4. PEAC modeling	10.09	2.75	0.41**	0.18**	0.28**											
5. PEAC threat	21.85	4.93	0.34**	0.29**	0.22**	0.45**										
Time 2																
6. SCARED-R Anxiety	15.38	7.97	0.62**	0.23**	0.38**	0.23**	0.28**									
7. IST C-bias	7.07	3.80	0.19**	0.42**	0.20**	0.02	0.17*	0.32**								
8. Amb Stories I-bias	5.03	3.23	0.22**	0.05	0.42**	0.08	0.14*	0.33**	0.26**							
9. PEAC modeling	9.94	2.77	0.29**	0.02	0.19**	0.41**	0.23**	0.36**	0.10	0.14*						
10. PEAC threat	21.67	5.04	0.19**	0.07	0.11	0.28**	0.51**	0.42**	0.18**	0.18*	0.52**					
Time 3																
11. SCARED-R Anxiety	14.69	8.50	0.56**	0.24**	0.48**	0.26**	0.19**	0.70**	0.20**	0.37**	0.30**	0.28**				
12. IST C-bias	8.41	3.71	0.23**	0.53**	0.28**	0.13	0.11	0.27**	0.61**	0.18**	0.08	0.08	0.30**			
13. Amb Stories I-bias	5.26	3.39	0.27**	0.17*	0.50**	0.09	0.11	0.29**	0.27**	0.47**	0.08	0.06	0.49**	0.35**		
14. PEAC modeling	9.85	2.80	0.27**	0.11	0.14*	0.41**	0.27**	0.30**	0.13	0.16*	0.50**	0.43**	0.33**	0.21**	0.17*	
15. PEAC threat	21.96	4.75	0.20**	0.16*	0.18**	0.18**	0.44**	0.36**	0.24**	0.27**	0.32**	0.55**	0.38**	0.21**	0.17**	0.47**

*SCARED-R* screen for child anxiety related disorders-revised, *IST* information search task, *C-bias* confirmation bias, *Amb Stories* ambiguous stories, *I-bias* interpretation bias, *PEAC* parental enhancement of anxious cognitions, *Threat* threat information transmission

*N* = *216*

*p < 0.05, **p < 0.01

### Correlations of the Parent-Report Data

In Table 2, an overview is given of the correlations between the PEAC scales as completed by the parents and both biases and anxiety as reported by the child. In general, these cross-informant correlations were weak and in most cases non-significant. The one exception was the correlation between parent-reported modeling and child-reported anxiety symptoms on time 2 (*r* = 0.20, *p* = 0.02). Given these findings, we decided to discard the parent data and merely focused on the child-report data in the remainder of the analyses.

**Table 2:** Correlations among the parent-reported PEAC scales and the child-reported anxiety and cognitive biases scores

	PEAC modeling parent	PEAC threat parent
Time 1		
SCARED-R Anxiety	0.04	0.13
IST C-bias	− 0.02	0.14
Amb Stories I-bias	0.04	0.02
Time 2		
SCARED-R Anxiety	0.20*	0.10
IST C-bias	0.04	0.08
Amb Stories I-bias	0.00	− 0.03
Time 3		
SCARED-R Anxiety	0.06	− 0.02
IST C-bias	− 0.05	0.04
Amb Stories I-bias	− 0.05	− 0.11

*SCARED-R* Screen for child anxiety related disorders-revised, *IST* information search task, *C-bias* confirmation bias, *Amb Stories* ambiguous stories, *I-bias* interpretation bias, *PEAC* parental enhancement of anxious cognitions, *Threat* threat information transmission

*N* = *147*

* *p* < 0.05

In passing, it should be noted that the parent–child correlations for the PEAC scales were also very low. For modeling, these correlations were 0.16 (*p* = 0.06) on time 1, 0.06 (*p* = 0.49) on time 2, and 0.21 (*p* = 0.01) on time 3. For threat information transmission, the correlations between parents and children were 0.16 (*p* = 0.05), 0.16 (*p* = 0.05), and 0.07 (*p* = 0.42) on the three subsequent time points.

### Cross-Sectional Models of the Relations Among Cognitive Biases and Anxiety

As can be seen in Table 3, the results indicated that models 3 and 5 provided the best fit for the cross-sectional relations among interpretation bias, confirmation bias, and anxiety. In the circular model (model 3), anxiety promoted interpretation bias, which had an enhancing effect on confirmation bias, which in turn again increased the level of anxiety. In model 5, both biases were the result of anxiety, with interpretation bias preceding confirmation bias. All models were subsequently re-run with equality constraints, that is, under the assumption that the path coefficient from X to Y is the same on all three time points for any given pair of variables X,Y (thereby reducing the number of path coefficients from 18 to 6 for the effects of age and gender on anxiety and biases in all models, from 9 to 3 for the paths among anxiety and both biases in models 3, 4, and 5, and from 6 to 2 for those paths in models 1 and 2). Likelihood Ratio (LR) testing of each constrained model against its unconstrained counterpart confirmed the validity of the equality constraints (see Table 3, the LR test uses the difference in Chi Square between both models as chi square test statistic, and the difference in degrees of freedom as the df for the LR test). Examination of the fit measures showed that models 3 and 5 again provided the best fit for the data. Because the circular model with bidirectional relations from bias to anxiety and vice versa (model 3) makes theoretically more sense [17] than a model in which biases are only by-products of anxiety (model 5), we chose model 3 with equality constraints as the starting point for further analyses.

**Table 3:**
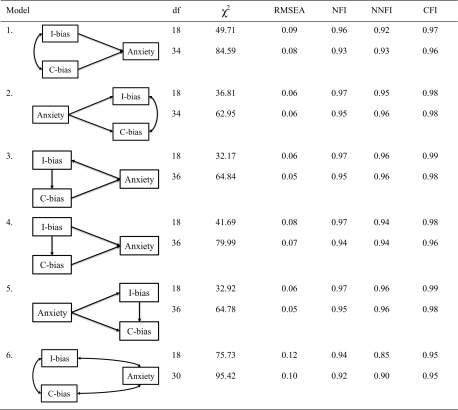
The six tested structural models of the cross-sectional relations among interpretation bias, confirmation bias, and anxiety symptoms on the three consecutive time points

The upper rows show the results of the analyses conducted *without* equality constraints and the lower rows the results of the analyses *with* equality constraints. *N* = *216*. One-way arrows reflect hypothesized to causal relations, while two-way arrows indicate correlation instead of effect. In all models, age and gender were included as exogenous variables having an effect on all psychological variables, and residual covariances were included between the repeated measures of a given psychological variable

*C-bias* confirmation bias, *I-bias* interpretation bias, *df* degrees of freedom, *RMSEA* root mean square of approximation, *NFI* Normed Fit Index, *NNFI* Non-normed Fit Index, *CFI* Comparative Fit Index

### Longitudinal Model of the Relations Among Cognitive Biases and Anxiety

**Fig. 1:**
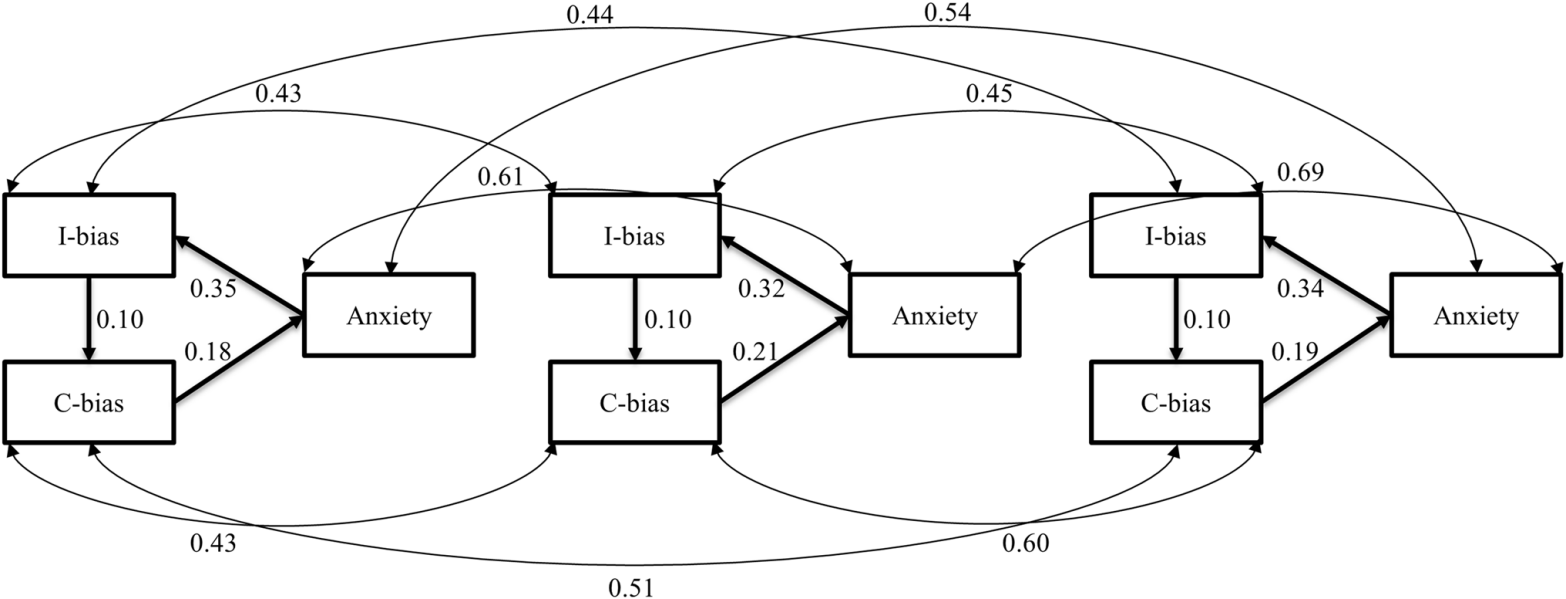
The best fitting model with circular relations among cognitive biases and anxiety on each of the three time points including residual correlations between the three repeated measurements of the same variable. Standardized path coefficients and residual correlations are shown. *C-bias* confirmation bias, *I-bias* interpretation bias. Equality constraints apply to unstandardized path coefficients. Standardized coefficients are not exactly equal across time points due to changes in variance of anxiety, C-bias, and I-bias over time. Age and gender are not displayed in the figure, although they were entered as exogenous variables with an effect on all psychological variables in the model

The following four extensions of model 3 with longitudinal paths were tested against model 3 with LR tests: (a) Time-lagged paths from both biases to anxiety; (b) Time-lagged paths from anxiety to both biases; (c) A combination of time-lagged paths from both biases to anxiety as well as time-lagged paths from anxiety to both biases; and (d) The same time-lagged paths as cross-sectional paths, that is, from anxiety to interpretation bias, from interpretation bias to confirmation bias, and from confirmation bias to anxiety. None of these four model extensions with time-lagged paths provided a better fit than model 3 with cross-sectional paths between the three constructs and residual correlations between the repeated measurements of a given constructs (i.e., anxiety, interpretation bias, confirmation bias). In all cases, the change in Chi Square was very close to the change in degrees of freedom and thus non-significant. Given the already very good fit of model 3 (see Table 3), this did not come as a complete surprise. Figure 1 shows the longitudinal version of model 3 including children’s anxiety and cognitive biases variables as measured on the three consecutive time points.

### Final model with parenting variables

The effects of the parenting variables of modeling and threat information transmission were first explored cross-sectionally by adding relations between parenting variables to the best fitting, theoretically most meaningful model (i.e., model 3) on each of the three time points. Three variants were tested: (a) A model in which parenting variables had an effect on anxiety and both cognitive biases; (b) A reversed model in which anxiety and cognitive biases had an impact on both parenting variables; and (c) A third and final model with bidirectional relations between parenting variables on the one hand and anxiety and cognitive biases on the other hand. In all three models we also assumed a cross-sectional correlation between modeling and threat information transmission as well as longitudinal correlations between the repeated measures of any given variable (e.g., between modeling at t and t + 1), and again we imposed equality constraints on all three paths for a given pair of variables (e.g., on the paths from modeling to anxiety at t1, t2, and t3).

Model C with bidirectional relations between parenting and anxiety/cognitive biases gave warnings and did not converge (probably due to unidentifiability). Model B with paths from anxiety and cognitive biases towards parenting variables had a better fit (*χ*^2^ = 169.76, df = 98, RMSEA = 0.05, NFI = 0.93, NNFI = 0.96, CFI = 0.97) than model A with paths from parenting variables to anxiety and cognitive biases (*χ*^2^ = 199.00, df = 98, RMSEA = 0.06, NFI = 0.92, NNFI = 0.94, CFI = 0.96).

**Fig. 2:**
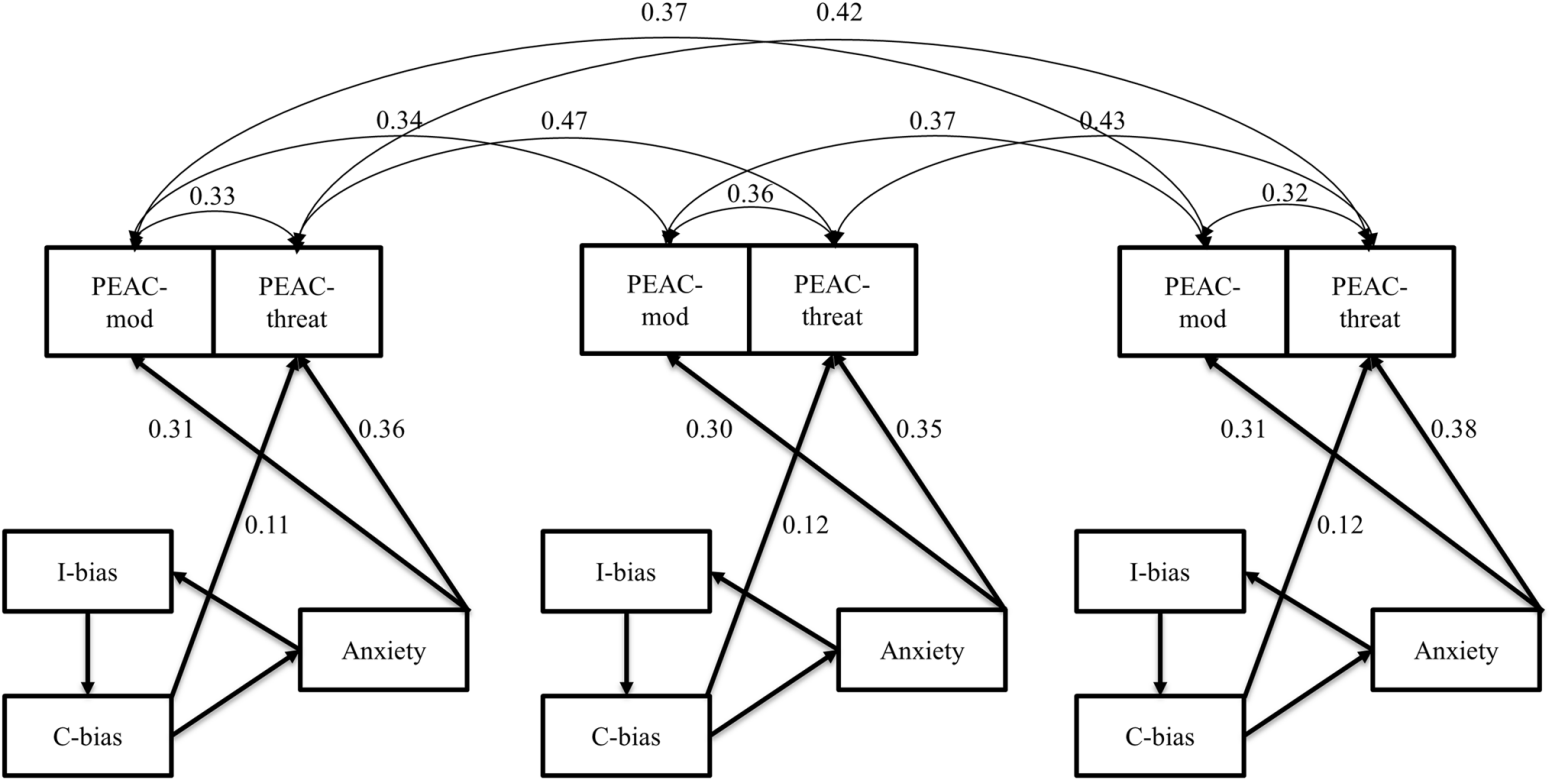
The best fitting model with circular relations among cognitive biases and anxiety on each of the three time points as well as paths from biases and anxiety towards both PEAC subscales. Standardized path coefficients are shown as well as residual correlations between the three repeated measures of the same PEAC variable and between modeling and threat information transfer at the same time point. *C-bias* confirmation bias, *I-bias* interpretation bias, *mod* modeling, *threat* threat information transmission. Equality constraints hold for the unstandardized path coefficients, but not for the residual variances, and thereby not exactly for the standardized path coefficients. Age and gender are not displayed in the figure, although they were entered as exogenous variables with an effect on all psychological variables in the model. For clarity reasons, non-significant paths as well as the residual correlations that are included in Fig. 1 are not shown in the figure, although they were included into the model

Extending model B with time-lagged paths from child to parenting variables or vice versa (e.g., from threat information transmission at t to anxiety at t + 1) did not improve the model fit and revealed no significant time-lagged effects. The best final model is therefore the model depicted in Fig. 2, with equality constraints and thus stability over time of all path coefficients.

### Mediation model

Note that we did not formally test a model in which cognitive biases acted as mediators in the relation between parental variables and children’s anxiety symptoms. We considered testing such a model as no longer appropriate, because we obtained no convincing evidence for a unidirectional relation between cognitive biases and anxiety (see Table 3).

## Discussion

The aim of the current study was to investigate associations among fear-enhancing parenting behaviors (modeling and threat information transmission), cognitive biases (interpretation bias and confirmation bias), and anxiety symptoms in non-clinical children, using a longitudinal study design consisting of three time points. Structural equation modeling by means of LISREL was employed to first test various models assuming only cross-sectional relations, and varying the direction of the paths among child anxiety, interpretation bias, and confirmation bias. Next, the best fitting, theoretically most plausible model was extended with longitudinal (cross-lagged) paths between anxiety and both biases. Finally, the parenting variables were added to the model. The key findings of the study can be catalogued as follows. First, we found support for a circular model in which cognitive biases promoted anxiety symptoms, which in turn enhanced cognitive biases. Second, no evidence was obtained for longitudinal effects indicating that cognitive biases increased anxiety levels or that anxiety strengthened cognitive biases over time. Third, the effects regarding fear-enhancing parenting were not completely as anticipated: the data suggested that cognitive biases and anxiety promoted modeling and negative information transmission rather than the other way around. Based on a previous cross-sectional analysis of these data (collected on time point 1; [39]) and current theoretical notions [28], we also expected to find support for a longitudinal mediational model in which cognitive biases would act as connector (mediator) between fear-enhancing parenting behaviors and children’s anxiety symptoms. However, the present findings indicated that there appeared to be reciprocal relations between cognitive biases and child anxiety and that child anxiety had an impact on parenting variables rather than the other way around. For these reasons, we considered tests of the hypothesized meditational model as no longer justifiable.

The analyses showed that children’s anxiety symptoms were significantly and positively associated with interpretation and confirmation bias on each of three assessment occasions. This is well in line with previous studies showing that anxious children display a stronger tendency to interpret ambiguous situations in a threatening way [12–14] and are more inclined to search for information that confirms threat [17–20]. LISREL analyses modeling the direction of the relations among anxiety and both biases revealed acceptable fits for most models. However, there were two models that stood out and displayed the best goodness-of-fit values across various fit indices. In one model, there was a unidirectional link from anxiety symptoms to both types of cognitive biases, whereas the other model was circular in nature with a bidirectional relation between anxiety and biases. Although there is certainly evidence showing that anxiety can precede the occurrence of cognitive biases, a wide range of studies have also demonstrated a reversed scenario in which these biases come first and contribute to the development of anxiety symptoms (see reviews by [54, 55]). A recent investigation by Remmerswaal et al. [17] demonstrated that in children such a bidirectional relation between cognitive biases and anxiety is also applicable, and for this reason we consider the circular, bidirectional model as theoretically more plausible than the model in which biases are just a by-product of anxiety.

An additional remark concerns the relation between cognitive biases and anxiety. The best fitting models had in common that there was a temporal order for both cognitive biases in which interpretation bias preceded confirmation bias. This sequence makes sense because interpretation bias describes the process of transforming a neutral or even an apparently positive event into a dangerous one and as such is typically considered to be the starting point of conscious threat perception [9]. In contrast, confirmation bias only occurs after threat has been perceived: the individual perceives the danger and subsequently searches for further information that confirms this threat [18].

In contrast to the support for relations between anxiety and cognitive biases, no cross-lagged, longitudinal relations were found. That is, no indications were found showing that (a) anxiety symptoms increased cognitive biases on subsequent time points, or that (b) cognitive biases enhanced anxiety symptoms over time. These results are in keeping with Muris et al. [56] who also failed to document prospective links between interpretation bias and children’s anxiety symptoms, but are obviously in contrast with Dodd et al. [57] who did show that threat interpretation predicted anxiety symptoms at a 12-month follow-up, and Creswell and O’Connor [58] who noted that anxiety symptoms predicted change in interpretation bias over time. Given these inconsistent findings, one might conclude that the contribution of cognitive biases occurring during the later stages of information processing, such as interpretation bias and confirmation bias, do not play a prominent role in the development of childhood anxiety symptoms. However, as the research so far has been mainly focused on young people in primary and middle childhood, it is still possible that this conclusion is only appropriate for early developmental stages and that the contribution of these cognitive biases to anxiety pathology becomes more important during adolescence and adulthood [59]. Obviously, replication of the present study in a sample of older youth is necessary to further investigate this possibility. Furthermore, it would be interesting to explore the link between cognitive biases and anxiety in young people who face a stressful life event or in at-risk children and adolescents of parents with anxiety disorders. As described in the introduction, previous research has shown that especially the offspring of parents with PD are more likely to show an interpretation bias [37, 38], and it would be particularly relevant to explore whether parental modeling and threat information transmission are involved in the familial transmission of this cognitive susceptibility.

Our analyses also showed substantial stability for both the cognitive biases and anxiety symptoms across the three assessment points. This means that the inter-individual variation in anxiety symptoms and associated cognitive biases did not show substantial changes during the year that the children were followed. The most important practical implication of this finding is that there seems to be a reliably identifiable subgroup of children with continuing high levels of anxiety symptoms that also show the typical concomitant cognitive features of this type of psychopathology (see also [60, 61]). It seems likely that these children are prone to develop a full-blown anxiety disorder when confronted with stress and adversity [62]. They probably constitute a suitable target population for prevention and early intervention efforts [4].

With regard to the parental variables of modeling and threat information transmission, our main prediction was that these parenting behaviors would enhance children’s anxiety symptoms and cognitive biases. The results first of all indicated that a model in which the parenting variables of modeling and threat information transmission had an enhancing effect on children’s anxiety symptoms and associated biases fitted the data rather well. However, the structural equations modeling analyses also revealed that a model in which anxiety symptoms and cognitive biases promoted fear-enhancing parental behaviors even had a slightly better fit. In view of these findings, a model with bidirectional relations between fear-enhancing parenting behaviors and children’s anxiety symptoms/cognitive biases seemed most plausible, but unfortunately that model appeared to be unidentifiable. Altogether, these results warrant the conclusion that a scenario in which anxiety symptoms and cognitive biases in children elicit anxious parenting is at least as likely as one in which fear-enhancing parenting elicits cognitive biases and anxiety. In the literature, several scholars have noted that fear-enhancing parental behaviors such as modeling and threat information transmission may intensify cognitive biases and anxiety symptoms in young people [32], but at the same time it is also possible that fear and anxiety symptomatology in children will evoke this type of parental behaviors in an attempt to help youngsters to face potentially threatening stimili and situations [40]. Meanwhile, it should be kept in mind that the above described findings regarding the role of parenting were mainly based on the analysis of children’s self-report data. It is possible that high-anxious children more easily perceive fear-promoting behaviors in their parents, which could also reflect some type of cognitive bias.

The present study also yielded a number of additional findings that need to be briefly discussed. First of all, for some variables, a significant gender effect was documented. In keeping with the literature, girls displayed higher levels of anxiety symptoms and interpretation bias than boys [51, 52]. Second, age was negatively related to anxiety symptoms and cognitive biases [50, 63, 64]. Thus, with increasing age, children reported lower levels of anxiety symptoms and indicated decreased tendencies to interpret ambiguous situation as threatening (interpretation bias) and to search for information that confirms threat (confirmation bias). Given that there are no reasons to assume that the relations between anxiety, cognitive biases, and parenting are different for boys and girls, and the fact that we only included young people of middle childhood with a limited age range, we did not explore moderation effects of gender and age but rather controlled for these demographic variables in our analyses.

A strong point of this study was that we tested cross-sectional as well as time-lagged relations between anxiety symptoms, cognitive biases, and parenting variables, eventually selecting the model that was the best compromise between goodness of fit (not lacking any relevant and significant paths or correlations) and parsimony (imposing sensible equality constraints). However, the present investigation also suffers from a number of limitations. To begin with, the study focused on a limited set of variables (modeling, threat information transmission, cognitive biases) that might be relevant within the etiology of childhood anxiety problems, thereby neglecting other factors (e.g., temperament, conditioning, overprotective parenting, and insecure attachment, or even protective factors) that are involved in the development of this type of psychopathology [65]. Second, although we did include parent rating scales and children’s self-report measures, most constructs were only assessed using one informant (i.e., either child or parent). The measure that was administered to children and parents (PEAC questionnaire) did not yield fully converging results, and this highlights the importance of including multiple informants for all variables [66]. Third, although an attempt was made to include both parents in the study, the participation rate was clearly higher for mothers than for fathers. As we used averaged parent scores if both parents had participated, or the scores of the one available parent else, it should be borne in mind that mothers were overrepresented in the present data set. Fourth, parenting was only assessed via a rating scale; it would have been better if we had employed some kind of interview or observational method to assess the fear-enhancing parental behaviors of modeling and threat information transmission. Apart from the fact that such a multi-method approach is preferable, this would also give us the opportunity to study the validity of the PEAC more thoroughly. Fifth, the study was carried out with a sample of non-clinical young people in middle childhood, a developmental stage with fairly little socio-emotional turmoil. It would be interesting to conduct a similar study in clinically referred youth or children/adolescents who face a significant life event. Sixth, children’s PEAC scores for mothers and fathers were strongly correlated, which may be the result of the method of scoring each item simultaneously for both parents (father ratings had to be provided on the left side of the screen, while mothers were given on the right side). For future studies, it would be better to present the father and mother versions of this questionnaire serially instead of employing this type of parallel assessment. Finally, the task for measuring interpretation bias included only three vignettes (i.e., one vignette per anxiety type: i.e., social anxiety, generalized anxiety, and separation anxiety) on each time point, and so one could question the validity of this task. However, it is important to note that this bias was not assessed by only three items as each vignette actually contained 5 items. This means that the interpretation bias scores, and this was also true for confirmation bias scores, were based on a total of 15 items, a number which was considered as more than sufficient for measuring these anxiety-related constructs. Moreover, for practical reasons, the administration of more vignettes was not desirable because this would have substantially increased the overall testing time for the children.

In spite of these limitations, this study yields important information on the cross-sectional and prospective relations between parenting, cognitive biases, and childhood anxiety. While the longitudinal analysis provided no support for the idea that cognitive biases are important for the etiology of childhood anxiety disorders, the data at least showed that these biases were solid correlates of anxiety and may fuel symptoms on each time point separately. This means that cognitive biases may indeed be a feasible target for interventions that aim to decrease anxiety levels in children, an idea that is of course already widespread in cognitive behavior therapy (CBT; [67]). Besides regular cognitive restructuring, an alternative option would be to apply bias modification approaches [68, 69] to undermine the cognitive biases to ultimately decrease the anxiety level. With regard to parenting, implications for therapy are less clear-cut: it seems common sense that parents should try not to increase their offspring’s anxiety symptoms by continuously modeling fear reactions or by constant communication of threat information. In the meantime, we should not overrate the importance of these parenting behaviors for the maintenance of children’s anxiety symptoms as they may just as likely be a reaction of the parents to an already anxious child. In support of this line of reasoning is the treatment literature which generally shows that adding parental components to CBT for anxious children does not necessarily imply that the intervention will be more effective [70]. Taken together, this research challenges a number of common assumptions on the etiology of childhood anxiety that are certainly a topic of further inquiry.

## Summary

Threat-related cognitive biases as well as fear-enhancing parenting behaviors are assumed to be involved in the etiology and maintenance of anxiety pathology in children. This longitudinal study explored whether (1) parental modeling and threat information transmission would be positively related to anxiety symptoms in children, and (2) if this relationship was mediated by children’s cognitive biases. On three subsequent time points over a one-year period, 7- to 12-year-old children and parents completed the Parental Enhancement of Anxious Cognitions scale, which measures parental modeling and threat information transmission, while children also completed a questionnaire of anxiety disorders symptoms and two computerized tasks measuring confirmation and interpretation bias. Results indicated that relations between cognitive biases and children’s anxiety symptoms were circular in nature. Thus, cognitive biases appeared to enhance anxiety symptoms, which in turn promoted cognitive biases on each of the three time points. No evidence was found for longitudinal effects of cognitive biases on anxiety or vice versa. The findings regarding the fear-enhancing parenting behaviors did not confirm our a priori hypothesis: cognitive biases and anxiety were found to promote parental modeling and threat information rather than the other way around. Because of these results, the mediation analysis of cognitive biases in the relation between fear-enhancing parenting and children’s anxiety symptoms was no longer justifiable. These findings provide further insight in the role of fear-enhancing parenting behaviors and cognitive biases in childhood anxiety.

## Footnotes

1 The finding that the drop-outs were significantly older probably has to do with the fact that the older children were in transition from primary to secondary school at the time of the second follow-up assessment. It was no longer possible to test these children ‘live’ at school, but they were asked to complete the measurements via the internet. Note that we adjusted for age (and gender) in the statistical analyses, so it is unlikely that this had a significant impact on the obtained results.

## References

[1] Polanczyk GV, Salum GA, Sugaya LS, Caye A, Rohde LA (2015). Annual Research Review: A meta-analysis of the worldwide prevalence of mental disorders in children and adolescents. J Child Psychol Psychiatry.

[2] Ford T, Goodman R, Meltzer H (2003). The British child and adolescent mental health survey 1999: the prevalence of DSM-IV disorders. J Am Acad Child Adolesc Psychiatry.

[3] Costello E, Mustillo S, Erkanli A, Keeler G, Angold A (2003). Prevalence and development of psychiatric disorders in childhood and adolescence. Arch Gen Psychiatry.

[4] Rapee RM, Schniering CA, Hudson JL (2009). Anxiety disorders during childhood and adolescence: origins and treatment. Annu Rev Clin Psychol.

[5] Muris P, Broeren S (2009). Twenty-five years of research on childhood anxiety disorders: Publication trends between 1982 and 2006 and a selective review of the literature. J Child Fam Stud.

[6] Beck AT (1976). Cognitive therapy and the emotional disorders.

[7] Harvey AG, Watkins E, Mansell W, Shafran R (2004). Cognitive behavioural processes across psychological disorders: a transdiagnostic approach to research and treatment.

[8] Hadwin JA, Field AP (2010). Information processing biases and anxiety: a developmental perspective.

[9] Muris P, Field AP (2008). Distorted cognition and pathological anxiety in children and adolescents. Cogn Emot.

[10] Daleiden EL, Vasey MW (1997). An information-processing perspective on childhood anxiety. Clin Psychol Rev.

[11] Muris P, Field A, Essau CA, Ollendick TH (2013). Information processing biases. The Wiley-Blackwell handbook of the treatment of childhood and adolescent anxiety.

[12] Muris P, Luermans J, Merckelbach H, Mayer B (2000). “Danger is lurking everywhere”. The relation between anxiety and threat perception abnormalities in normal children. J Behav Ther Exp Psychiatry.

[13] Bögels SM, Zigterman D (2000). Dysfunctional cognitions in children with social phobia, separation anxiety disorder, and generalized anxiety disorder. J Abnorm Child Psychol.

[14] Bell-Dolan DJ (1995). Social cue interpretation of anxious children. J Clin Child Psychol.

[15] Cannon MF, Weems CF (2010). Cognitive biases in childhood anxiety disorders: do interpretive and judgment biases distinguish anxious youth from their non-anxious peers?. J Anxiety Disord.

[16] Waters AM, Wharton TA, Zimmer-Gembeck MJ, Craske MG (2008). Threat-based cognitive biases in anxious children: comparison with non-anxious children before and after cognitive behavioural treatment. Behav Res Ther.

[17] Remmerswaal D, Huijding J, Bouwmeester S, Brouwer M, Muris P (2014). Cognitive bias in action: evidence for a reciprocal relation between confirmation bias and fear in children. J Behav Ther Exp Psychiatry.

[18] Muris P, Rassin E, Mayer B, Smeets G, Huijding J, Remmerswaal D (2009). Effects of verbal information on fear-related reasoning biases in children. Behav Res Therapy.

[19] Dibbets P, Meesters C (2017). The influence of stimulus valence on confirmation bias in children. J Behav Ther Exp Psychiatry.

[20] Dibbets P, Fliek L, Meesters C (2015). Fear-related confirmation bias in children: a comparison between neutral-and dangerous-looking animals. Child Psychiatry Hum Dev.

[21] Pérez-Edgar K, Bar-Haim Y, McDermott JM, Gorodetsky E, Hodgkinson CA, Goldman D (2010). Variations in the serotonin-transporter gene are associated with attention bias patterns to positive and negative emotion faces. Biol Psychol.

[22] Thomason ME, Henry ML, Hamilton JP, Joormann J, Pine DS, Ernst M (2010). Neural and behavioral responses to threatening emotion faces in children as a function of the short allele of the serotonin transporter gene. Biol Psychol.

[23] Zavos HM, Rijsdijk FV, Gregory AM, Eley TC (2010). Genetic influences on the cognitive biases associated with anxiety and depression symptoms in adolescents. J Affect Disord.

[24] Murray L, Creswell C, Cooper PJ (2009). The development of anxiety disorders in childhood: an integrative review. Psychol Med.

[25] Askew C, Field AP (2008). The vicarious learning pathway to fear 40 years on. Clin Psychol Rev.

[26] Muris P, Field AP (2010). The role of verbal threat information in the development of childhood fear. “Beware the Jabberwock!”. Clin Child Fam Psychol Rev.

[27] Creswell C, Cooper P, Murray L, Hadwin JA, Field AP (2010). Intergenerational transmission of anxious information processing biases. Information processing biases and anxiety. A developmental perspective.

[28] Hadwin JA, Garner M, Perez-Olivas G (2006). The development of information processing biases in childhood anxiety: a review and exploration of its origins in parenting. Clin Psychol Rev.

[29] Last CG, Hersen M, Kazdin A, Orvaschel H, Perrin S (1991). Anxiety disorders in children and their families. Arch Gen Psychiatry.

[30] Lester KJ, Field AP, Cartwright-Hatton S (2012). Maternal anxiety and cognitive biases towards threat in their own and their child’s environment. J Fam Psychol.

[31] Creswell C, O’Connor TG, Brewin CR (2006). A longitudinal investigation of maternal and child ‘anxious cognitions’. Cognit Therapy Res.

[32] Fisak B, Grills-Taquechel A (2007). Parental modeling, reinforcement, and information transfer: risk factors in the development of child anxiety?. Clin Child Fam Psychol Rev.

[33] Wood JJ, McLeod BD, Sigman M, Hwang WC, Chu BC (2003). Parenting and childhood anxiety: theory, empirical findings, and future directions. J Child Psychol Psychiatry.

[34] Barrett PM, Rapee RM, Dadds MM, Ryan SM (1996). Family enhancement of cognitive style in anxious and aggressive children. J Abnorm Child Psychol.

[35] Sicouri G, Sharpe L, Hudson JL, Dudeney J, Jaffe A, Selvadurai H (2017). Threat interpretation and parental influences for children with asthma and anxiety. Behav Res Ther.

[36] Remmerswaal D, Muris P, Huijding J (2016). Transmission of cognitive bias and fear from parents to children: an experimental study. Journal of Clinical Child Adolescent Psychology.

[37] Van Niekerk RE, Klein AM, Allart-van Dam E, Rinck M, Souren PM, Hutschemaekers GJM (2018). Biases in interpretation as a vulnerability factor for children of parents with an anxiety disorder. J Am Acad Child Adolesc Psychiatry.

[38] Schneider S, Unnewehr S, Florin I, Margraf J (2002). Priming panic interpretations in children of patients with panic disorder. J Anxiety Disord.

[39] Fliek L, Dibbets P, Roelofs J, Muris P (2017). Cognitive bias as a mediator in the relation between fear-enhancing parental behaviors and anxiety symptoms in children: a cross-sectional study. Child Psychiatry Hum Dev.

[40] Rapee RM (2009). Early adolescents’ perceptions of their mother’s anxious parenting as a predictor of anxiety symptoms 12 months later. J Abnorm Child Psychol.

[41] Muris P, Merckelbach H, Van Brakel A, Mayer B (1999). The revised version of the screen for child anxiety related emotional disorders (SCARED-R): further evidence for its reliability and validity. Anxiety Stress Coping.

[42] Birmaher B, Khetarpal S, Brent D, Cully M, Balach L, Kaufman J (1997). The Screen for Child Anxiety Related Emotional Disorders (SCARED): scale construction and psychometric characteristics. J Am Acad Child Adolesc Psychiatry.

[43] American Psychiatric Association (2000). Diagnostic and statistical manual of mental disorders.

[44] American Psychiatric Association (1994). Diagnostic and statistical manual of mental disorders.

[45] Muris P, Dreessen L, Bögels SM, Weckx M, Van Melick M (2004). A questionnaire for screening a broad range of DSM-defined anxiety disorder symptoms in clinically referred children and adolescents. J Child Psychol Psychiatry.

[46] Muris P, Merckelbach H, Schmidt H, Mayer B (1999). The revised version of the Screen for Child Anxiety Related Emotional Disorders (SCARED-R): factor structure in normal children. Personality Individ Differ.

[47] Muris P, Rapee R, Meesters C, Schouten E, Geers M (2003). Threat perception abnormalities in children: the role of anxiety disorders symptoms, chronic anxiety, and state anxiety. J Anxiety Disord.

[48] Joreskog K, Sorbom D (1989). LISREL 7: user’s reference guide.

[49] Joreskog K, Sorbom D (1993). LISREL 8: manual.

[50] Westenberg PM, Siebelink BM, Warmenhoven NJC, Treffers PDA (1999). Separation anxiety and overanxious disorders: relations to age and level of psychosocial maturity. J Am Acad Child Adolesc Psychiatry.

[51] Lewinsohn PM, Gotlib IH, Lewinsohn M, Seeley JR, Allen NB (1998). Gender differences in anxiety disorders and anxiety symptoms in adolescents. J Abnorm Psychol.

[52] Craske MG (2003). Origins of phobias and anxiety disorders: why more women than men.

[53] Browne MW, Cudeck R, Bollen KA, Long JS (1993). Alternative ways of assessing model fit. Testing structural equation models.

[54] Wilson EJ, MacLeod C, Mathews A, Rutherford EM (2006). The causal role of interpretive bias in anxiety reactivity. J Abnorm Psychol.

[55] Van Bockstaele B, Verschuere B, Tibboel H, De Houwer J, Crombez G, Koster EHW (2014). A review of current evidence for the causal impact of attentional bias on fear and anxiety. Psychol Bull.

[56] Muris P, Jacques P, Mayer B (2004). The stability of threat perception abnormalities and anxiety disorder symptoms in non-clinical children. Child Psychiatry Hum Dev.

[57] Dodd HF, Hudson JL, Morris TM, Wise CK (2012). Interpretation bias in preschool children at risk for anxiety: a prospective study. J Abnorm Psychol.

[58] Creswell C, O’Connor TG (2011). Interpretation bias and anxiety in childhood: stability, specificity and longitudinal associations. Behavioural Cognitive Psychotherapy.

[59] Creswell C, Murray L, Cooper P (2014). Interpretation and expectation in childhood anxiety disorders: age effects and social specificity. J Abnorm Child Psychol.

[60] Ialongo N, Edelsohn G, Werthamer-Larsson L, Crockett L, Kellam S (1995). The significance of self-reported anxious symptoms in first grade children: prediction to anxious symptoms and adaptive functioning in fifth grade. J Child Psychol Psychiatry.

[61] Bosquet M, Egeland B (2006). The development and maintenance of anxiety symptoms from infancy through adolescence in a longitudinal sample. Dev Psychopathol.

[62] Muris P (2007). Normal and abnormal fear and anxiety in children and adolescents.

[63] Duchesne S, Vitaro F, Larose S, Tremblay RE (2008). Trajectories of anxiety during elementary-school years and the prediction of high school noncompletion. J Youth Adolesc.

[64] Broeren S, Muris P, Diamantopoulou S, Baker JR (2013). The course of childhood anxiety symptoms: developmental trajectories and child-related factors in normal children. J Abnorm Child Psychol.

[65] Vasey MW, Dadds MR, Vasey MW, Dadds MR (2001). An introduction to the developmental psychopathology of Anxiety. The developmental psychopathology of anxiety.

[66] De Los Reyes A, Kazdin AE (2005). Informant discrepancies in the assessment of childhood psychopathology: a critical review, theoretical framework, and recommendations for further study. Psychol Bull.

[67] Kendall PC (1993). Cognitive-behavioral therapies with youth: guiding theory, current status, and emerging developments. J Consult Clin Psychol.

[68] Reuland MM, Teachman BA (2014). Interpretation bias modification for youth and their parents: a novel treatment for early adolescent social anxiety. J Anxiety Disord.

[69] Amir N, Beard C, Burns M, Bomyea J (2009). Attention modification program in individuals with generalized anxiety disorder. J Abnorm Psychol.

[70] Bodden DHM, Bögels SM, Nauta MH, De Haan E, Ringrose J, Appelboom C (2008). Child versus family cognitive-behavioral therapy in clinically anxious youth: an efficacy and partial effectiveness study. J Am Acad Child Adolesc Psychiatry.

